# Clinical implication of serum biomarkers and patient age in inflammatory demyelinating diseases

**DOI:** 10.1002/acn3.51070

**Published:** 2020-06-04

**Authors:** Eun‐Jae Lee, Young‐Min Lim, Seungmi Kim, Lynkyung Choi, Hyunjin Kim, Keonwoo Kim, Hye Weon Kim, Ji Sung Lee, Kwang‐Kuk Kim

**Affiliations:** ^1^ Department of Neurology Asan Medical Center University of Ulsan College of Medicine Seoul South Korea; ^2^ Department of Medicine Asan Medical Institute of Convergence Science and Technology, University of Ulsan College of Medicine Seoul South Korea; ^3^ Clinical Research Center Asan Institute for Life Sciences Asan Medical Center University of Ulsan College of Medicine Seoul South Korea

## Abstract

**Objectives:**

Serum synaptic proteins levels may change with age‐related neurodegeneration, affecting their clinical implications as a disease biomarker. We aimed to investigate neuronal and astroglial markers in patients with multiple sclerosis (MS) and aquaporin‐4 antibody‐seropositive neuromyelitis optica spectrum disorders (NMOSD) to compare the clinical implications of these markers according to age.

**Methods:**

Using single‐molecule array assays, we measured neurofilament light (NfL) and glial fibrillary acidic protein (GFAP) in sera from consecutive patients with MS (n = 117) and NMOSD (n = 63). For each disease, we assessed correlations between these markers and disease severity (Expanded Disability Status Scale [EDSS]) scores according to three age groups (≤44, 45–54, and ≥55 years).

**Results:**

Although serum GFAP levels were significantly higher in patients with NMOSD than those with MS, levels of both serum markers revealed significant positive correlations with EDSS scores in both diseases. In MS patients, the degrees of correlation between serum NfL (or GFAP) levels and EDSS scores were similar across all age groups. However, in NMOSD patients, positive GFAP‐EDSS correlations were distinctively stronger in the youngest than in the oldest group. Conversely, there were no positive NfL‐EDSS correlations in NMOSD in the youngest group, but there were significant in the oldest group.

**Interpretation:**

The degrees to which serum NfL and GFAP levels reflect disease severity vary significantly with patient age in NMOSD, but not in MS. These findings suggest that the pathological processes and progression differ between the diseases; hence, serum biomarker levels may need to be interpreted differently according to patient age and disease type.

## Introduction

Multiple sclerosis (MS) and neuromyelitis optica spectrum disorders (NMOSD) are chronic inflammatory demyelinating diseases of the central nervous system.^1^ These disorders are characterized by relapses and deterioration, which necessitates biomarkers for long‐term disease monitoring.^2^ With the recent development of ultrasensitive single‐molecule array (Simoa) technology,^3^ serum neurofilament light protein (NfL), a neuronal damage marker, and glial fibrillary acidic protein (GFAP), an astrocyte‐damage marker, have been suggested as a good biomarker candidate with high sensitivity.^4^, ^5^, ^6^, ^7^, ^8^, ^9^, ^10^ However, processes associated with aging, such as neurodegeneration and related astrogliosis may influence these protein biomarkers;^6^, ^11^, ^12^, ^13^, ^14^ thus, these proteins’ clinical implications may differ depending on patient age.

The pathogenic mechanisms and disease courses of MS and NMOSD are different.^1^ MS affects myelin and oligodendrocytes, the pathogenic antigen of which remains elusive, whereas NMOSD is an astrocytopathy that targets aquaporin‐4 (AQP4) protein in astrocytes.^1^ MS progression is largely independent of inflammatory relapsing activity; neurodegenerative processes that are separate from clinical relapsing events may be important in this disorder. Conversely, a progressive phase is rare in NMOSD and neurodegeneration in this disorder depends mainly on inflammatory relapses.^1^

We hypothesized that the clinical implications of serum protein markers associated with disease activity may change with age and their changing patterns may differ between MS and NMOSD. To this end, we investigated the serum levels of NfL and GFAP in consecutive patients with MS and NMOSD. We evaluated correlations between the levels of these biomarkers and disease severity in patients with MS and NMOSD and compared the degrees of correlation according to age within and between disease groups.

## Methods

### Patients

Consecutive patients with MS and NMOSD^15^ who visited the Department of Neurology at the Asan Medical Center (Seoul, Korea) were prospectively recruited between July 2018 and February 2019. Enrolled MS and NMOSD patients fulfilled the 2017 McDonald criteria^16^, ^17^ and the 2015 Wingerchuk criteria,^15^ respectively. All these patients underwent tests for antibodies against AQP4 protein and myelin oligodendrocyte glycoprotein (MOG) by a cell‐based assay. For the NMOSD group, we only included patients seropositive for anti‐AQP4 antibody; the absence of history of optic neuritis was not an exclusion criterion. We did not include patients who revealed anti‐MOG antibodies to promote our research purposes. The Expanded Disability Status Scale (EDSS) score was evaluated at the time of blood sampling. Our Institutional Review Board approved this study (No. 2018‐0653), and written informed consent was obtained from all participants.

### Serum sampling and analysis

All participants underwent Simoa analysis. Serum samples were collected and stored at −80℃ according to standardized procedures.^18^ Samples were thawed immediately before analysis. NfL and GFAP levels were measured in duplicate using a Simoa HD‐1 Analyzer (Quanterix, MA, USA) at PrismCDX (Gyeonggi‐do, Korea), by an investigator who was blinded to the clinical information.^19^, ^20^ Serum samples were diluted to 1:4 according to the Quanterix guideline and other study groups,^6^, ^7^, ^8^, ^9^, ^21^ while the quantification limit was 0.104 pg/mL for NfL, and 0.221 pg/mL for GFAP; all results were above the quantification limit. The mean intra‐assay coefficients of variation for the NfL and GFAP levels were 4.2% and 2.9%, respectively. All intra‐assay duplicate coefficients of variation for the samples were less than 20%.

### Statistical analysis

The chi‐squared or Mann–Whitney *U* test was performed to compare variables between MS and NMOSD patients; serum marker levels were log transformed to meet the normal assumption. An analysis of covariance was conducted to compare serum markers, adjusting for important clinical variables (age, EDSS score, days from the last attack to blood sampling, recent relapses <60 days) that may affect the serum marker levels. Pearson correlation coefficients were calculated to evaluate correlations among the log‐transformed levels of serum biomarkers within each disease group. Then, associations between these serum markers and clinical variables (age, disease duration, and EDSS score) were also calculated with Pearson correlation coefficients; age was used as a continuous variable in these analyses. Linear regression models were also applied to identify associations between EDSS scores and serum marker levels. In these models, regression coefficients (β) were back‐transformed to the original scale to reflect multiplicative effects.^6^, ^7^

To assess whether the clinical significance of serum markers would differ depending on patient age, we evaluated the degrees of association between the biomarkers and EDSS scores in three ways, according to the age groups. First, we analyzed the association by stratification of decade‐year groups (≤30, 31–40, 41–50, 51–60, ≥61 years). Next, to increase the number of patients in each group, we performed the analysis after classifying the patients into three age groups (≤44, 45–54, and ≥55 years) based on previous studies.^22^, ^23^, ^24^ Finally, we set up two age groups according to the median age to verify the results from the analysis of the three age groups. The degrees of correlation between the disease (or age) groups were compared using Fisher’s z‐transformation of correlation coefficients. We evaluated correlations between the disease (MS vs. NMOSD) groups and between the age (≤44 vs. 45‐54 vs. ≥55 years) groups. When correlations between the age groups were compared, the youngest group was referenced; a Bonferroni correction was performed to correct for multiple comparisons.

The period from the last clinical attacks to blood sampling was shorter in the NMOSD group than the MS group. Hence, we proceeded to age‐group analyses using only data from patients who had their last clinical attack within the previous 5 years to balance the variable. Significance was set at two‐tailed *P* < 0.05. All analyses were conducted using Stata version 13.0 (StataCorp., TX, USA).

### Data availability

The authors are willing to provide the anonymized data related to this work upon reasonable request.

## Results

During the study period, 119 patients met the McDonald criteria, while 63 patients fulfilled the Wingerchuk criteria with anti‐AQP4 antibodies. Of them, two patients in the former group had anti‐MOG antibodies, thus were not included in this analysis. A total of 180 patients (117 MS and 63 NMOSD) were finally enrolled (Table 1). The median age was 47 years and 142 (78.9%) patients were female. NMOSD patients were older than MS patients at the time of sampling and had more frequent relapses and recent attacks and a higher EDSS score. The period between the last clinical attack and blood sampling was shorter in the NMOSD group. NfL was quantified in 174 and GFAP in 168 patients. Serum NfL and GFAP levels were higher in NMOSD patients than MS patients; after adjusting for important clinical variables that may affect biomarker levels (age, EDSS score, days from the last attack to blood sampling, recent relapse <60 days), only GFAP levels remained significantly elevated in NMOSD patients.

**Table 1:** Baseline characteristics between patients with multiple sclerosis and those with seropositive (anti‐aquaporin‐4 antibody) neuromyelitis optica spectrum disorders.

	MS	NMOSD	*P*	*P* †
	(n = 117)	(n = 63)		
Age at present	45 [34–54]	54 [46–60]	<0.001	
Age at onset	33 [25–42]	44 [35–52]	<0.001	
Follow‐up, years	10 [5–13]	8 [3–14]	0.077	
Days from the last attack	1068 [387–2883]	633 [127–1311]	0.002	
Female	85 (72.6)	57 (90.5)	0.005	
No. of attacks	3 [1–4]	3 [2–5]	0.265	
No. of ON attacks	0 [0–1]	1 [0–2]	0.087	
No. of TM attacks	1 [0–2]	2 [1–3]	0.011	
No. of Brain attacks	1 [0–2]	0 [0–1]	0.021	
Recent relapse, <60 d	8 (6.9)	12 (19.0)	0.014	
Annual Relapse Rate	0.3 [0.2–0.6]	0.5 [0.3–0.8]	0.001	
Monophasic, n (%)	30 (25.6)	13 (20.6)	0.452	
Simultaneous attacks*	6 (5.1)	7 (11.1)	0.225	
(any time)				
EDSS, median (quartiles)	2.0 [1.0–4.0]	3.5 [2.0–5.0]	0.007	
Treatments				
Any immunomodulating agent	103 (88.0)	63 (100.0)	0.002	
Prednisolone	11 (9.4)	31 (49.2)	<0.001	
Azathioprine	0 (0.0)	32 (50.0)	<0.001	
Mycophenolate mofetil	2 (1.7)	11 (17.5)	<0.001	
Interferon‐β	33 (28.2)	0 (0.0)	<0.001	
Teriflunomide	31 (26.5)	0 (0.0)	<0.001	
Dimethyl fumarate	7 (6.0)	0 (0.0)	0.098	
Glatiramer acetate	4 (3.4)	0 (0.0)	0.289	
Fingolimod	7 (6.0)	0 (0.0)	0.054	
Alemtuzumab	12 (10.3)	0 (0.0)	0.009	
Rituximab	0 (0.0)	16 (25.4)	<0.001	
Simoa markers				
NfL (pg/mL)^‡^	10.2 [8.2–15.1] (n = 113)	14.6 [10.2–22.7] (n = 61)	0.001	0.356
GFAP (pg/mL)^‡^	95.0 [70.0–130.4] (n = 112)	126.8 [97.3–225.1] (n = 56)	<0.001	0.014

EDSS, Expanded Disability Severity Scale; GFAP, glial fibrillary acidic protein; MS, multiple sclerosis; NfL, neurofilament light chain; NMOSD, neuromyelitis optica spectrum disorders; ON, optic neuritis; TM, transverse myelitis.

* Simultaneous attacks of optic neuritis and transverse myelitis.

^†^ *P* value after adjustment for age, EDSS score, days from the last attack to blood sampling, recent relapse <60 days.

^‡^ Statistical analyses with these variables were performed with log‐transformed values.

**Figure 1:**
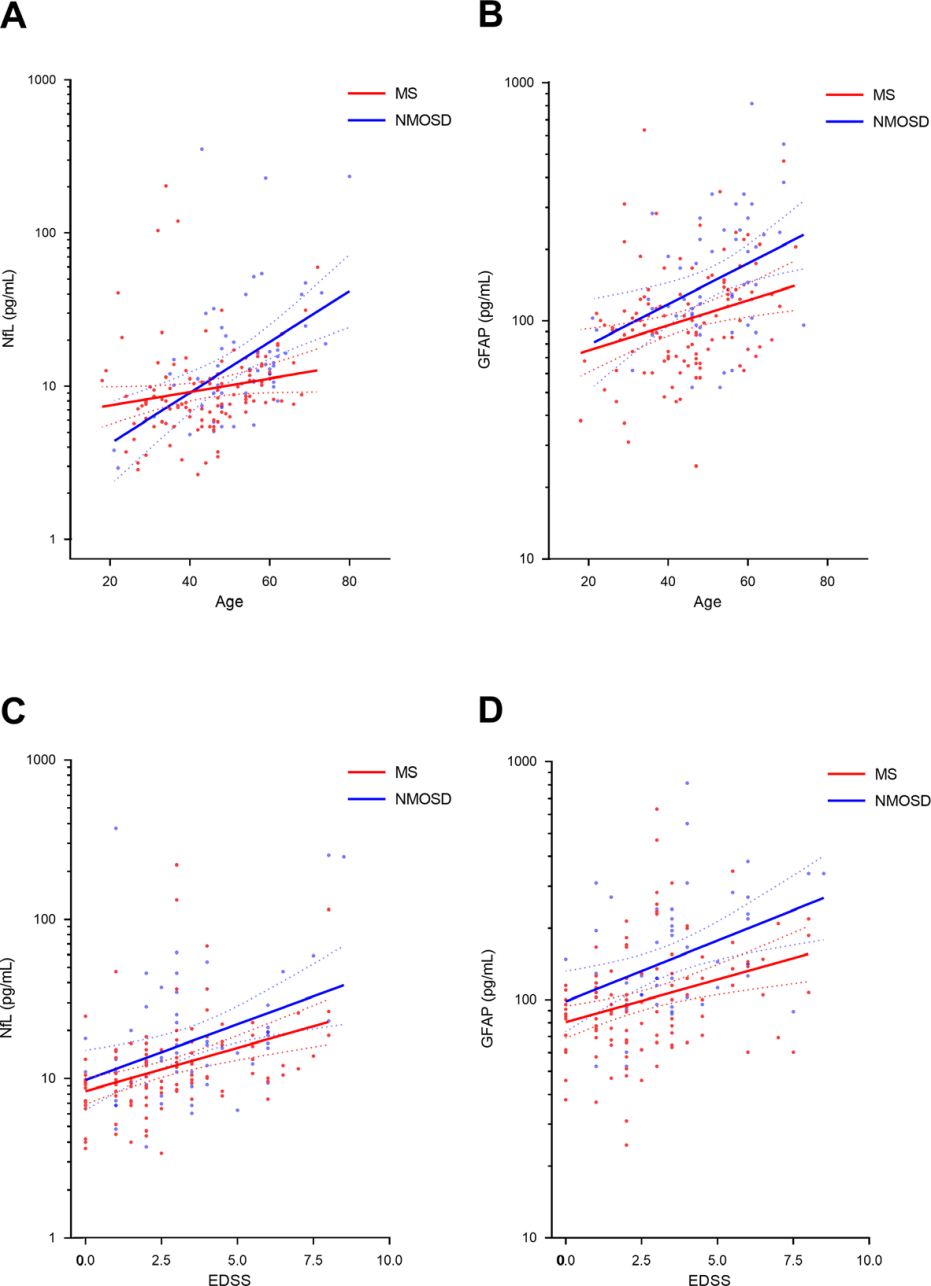
Associations between serum biomarkers and age and Expanded Disability Status Scale (EDSS) score. (A) Serum neurofilament light chain (NfL) levels and age (MS: β = 1.009, *P* = 0.060; NMOSD: β = 1.038, *P* < 0.001), (B) serum glial fibrillary acid protein (GFAP) levels and age (MS: β = 1.012, p = 0.003; NMOSD: β = 1.020, *P* = 0.004), (C) serum NfL levels and EDSS (MS: β = 1.134, p = 0.003; NMOSD: β = 1.175, *P* = 0.003), (D) serum GFAP levels and EDSS (MS: β = 1.086, p = 0.001; NMOSD: β = 1.125, *P* = 0.003). MS, multiple sclerosis; NMOSD, neuromyelitis optica spectrum disorders.

Serum GFAP and NfL levels were significantly and positively correlated with each other in both diseases (Pearson r = 0.550, *P* < 0.001 for MS; Pearson r = 0.346, *P* = 0.009 for NMOSD). Subsequently, we evaluated the correlations between serum marker levels and clinical variables within each disease group (Table 2). NfL levels showed significant positive correlations with age in NMOSD (Pearson r = 0.480, *P* < 0.001) but not MS (Pearson r = 0.177, *P* = 0.060). Moreover, positive NfL‐age correlations in NMOSD patients were more significant than those in MS patients (MS vs. NMOSD, *P* = 0.034; Fig. 1A). Meanwhile, GFAP levels increased significantly with age in both diseases (Fig. 1B). In terms of disease severity, both NfL and GFAP levels revealed significant positive correlations with EDSS scores in both diseases (Fig. 1C and D).

**Table 2:** Correlations between serum markers and clinical variables.

Correlation	MS		NMOSD		*P* ^†^
	(n = 117)		(n = 63)		
	Pearson r	n	Pearson r	n	
Age					
log NfL	0.177 (*P* = 0.060)	113	0.480*** (*P* < 0.001)	61	0.034
log GFAP	0.280** (*P* = 0.003)	112	0.380** (*P* = 0.004)	56	0.502
Disease duration					
log NfL	−0.074 *P* = 0.436)	113	−0.049 (*P* = 0.708)	61	0.877
log GFAP	0.076 (*P* = 0.423)	112	0.190 (*P* = 0.162)	56	0.488
EDSS					
log NfL	0.382*** (*P* < 0.001)	113	0.369** (*P* = 0.003)	61	0.926
log GFAP	0.317*** (*P* = 0.001)	112	0.396** (*P* = 0.003)	56	0.589

EDSS, expanded disability severity scale; GFAP, glial fibrillary acidic protein; MS, multiple sclerosis; NfL, neurofilament light chain; NMOSD, neuromyelitis optica spectrum disorders.

Significant correlations with age or EDSS sore,

* *P* < 0.05,

** *P* < 0.01,

*** *P* < 0.001;

^†^ *P* value for differences in correlation coefficients between the MS and NMOSD groups.

We then analyzed the correlations between the serum markers and EDSS scores according to the age groups. First, patients were classified according to the decade of age (Table 3). However, the number of patients in each age group was small; particularly that in the youngest NMOSD patients (≤30 years) was too small (n = 2) to determine the correlation coefficient. Although no significant interactions were identified across the age groups, GFAP levels in NMOSD patients showed significant positive correlations with EDSS scores in the younger age groups (31–40 and 41–50 years), while NfL levels showed significant positive correlations with EDSS scores in the older age groups (51–60 and ≥61 years).

**Table 3:** Correlations between serum markers and Expanded Disability Status Scale scores according to age (by decade).

	MS				NMOSD				*P* ^†^ (MS vs. NMOSD)	
	Pearson r	n	*P* ^‡^		Pearson r	n	*P* ^‡^		Uncorrected	Corrected (Bonferroni)
			Uncorrected	Corrected (Bonferroni)			Uncorrected	Corrected (Bonferroni)		
Log NfL										
≤30	−0.004	18			NA	2	NA	NA	NA	NA
31−40	0.526**	29	0.070	0.280	−0.124	7			0.187	0.935
41−50	0.327	32	0.280	>0.999	−0.330	18	0.698	>0.999	0.032	0.160
51−60	0.074	24	0.817	>0.999	0.568*	19	0.169	0.676	0.086	0.430
≥61	0.226	10	0.609	>0.999	0.567*	15	0.184	0.736	0.385	>0.999
Log GFAP										
≤30	0.315	18			NA	2	NA	NA	NA	NA
31−40	0.168	29	0.629	>0.999	0.740*	7			0.147	0.735
41−50	0.055	31	0.397	>0.999	0.589*	16	0.631	>0.999	0.064	0.320
51−60	0.421*	24	0.716	>0.999	0.300	18	0.255	>0.999	0.680	>0.999
≥61	0.058	10	0.558	>0.999	0.118	13	0.160	0.640	0.902	>0.999

EDSS, expanded disability severity scale; GFAP, glial fibrillary acidic protein; MS, multiple sclerosis; NA, not available; NfL, neurofilament light chain; NMOSD, neuromyelitis optica spectrum disorders.

Significant correlations with EDSS score,

* *P* < 0.05,

** *P* < 0.01,

*** *P* < 0.001;

^†^ *P* value for differences in correlation coefficients between the MS and NMOSD groups;

^‡^ *P* value for differences in correlation coefficients between the age groups within the disease group (vs. the reference, MS: ≤30 years, NMOSD: 31−40 years).

**Figure 2:**
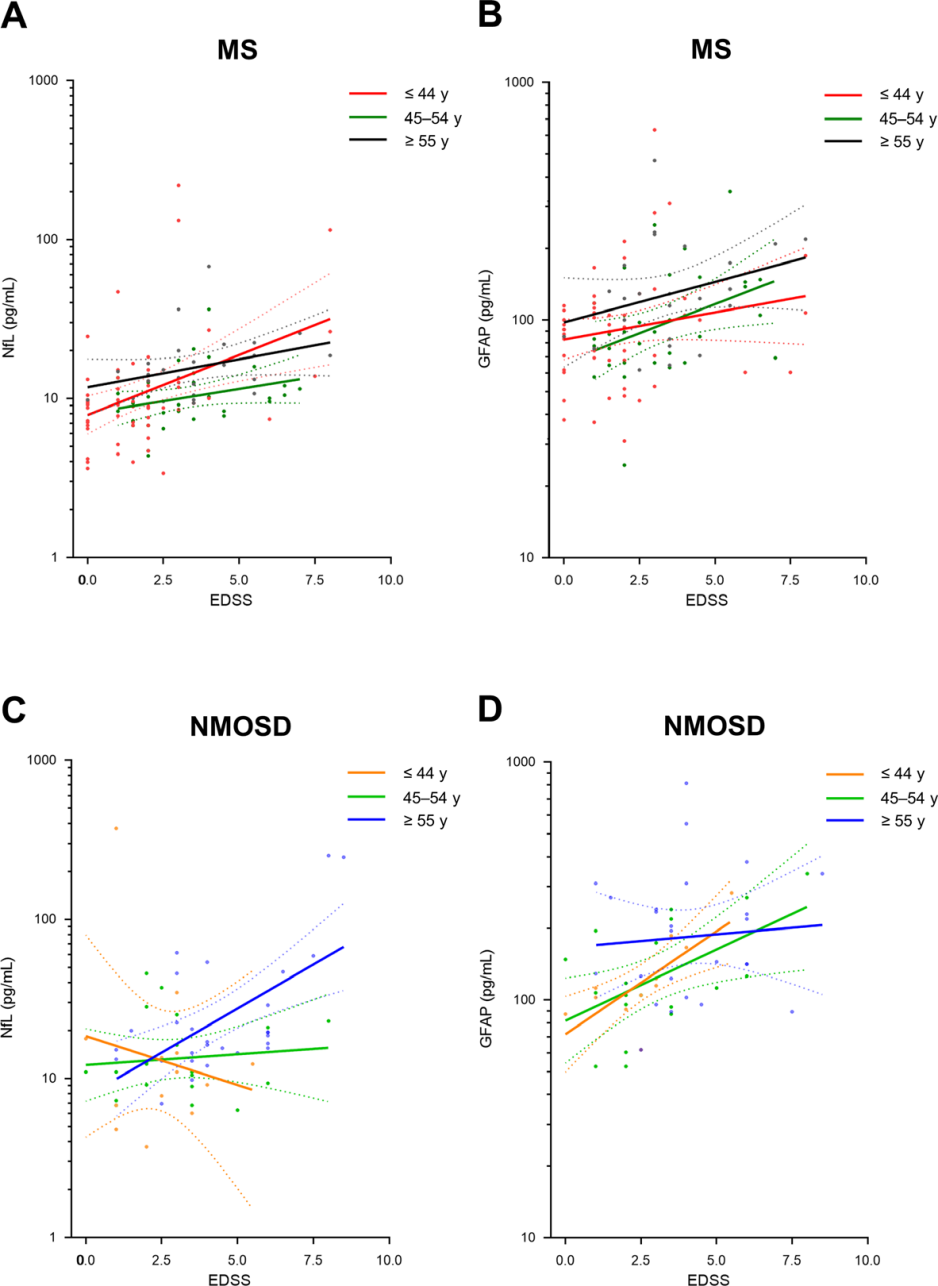
Associations between serum biomarkers and Expanded Disability Status Scale (EDSS) score according to three age groups. (A) Serum neurofilament light chain (NfL) levels and EDSS in MS patients (≤44 years: β = 1.191, *P* = 0.001; 45–54 years: β = 1.075, *P* = 0.094; ≥55 years: β = 1.085, *P* = 0.111), (B) serum glial fibrillary acid protein (GFAP) levels and EDSS in MS patients (≤44 years: β = 1.055, *P* = 0.149; 45–54 years: β = 1.118, *P* = 0.028; ≥ 55 years: β = 1.082, *P* = 0.142), (C) serum NfL levels and EDSS in NMOSD patients (≤44 years: β = 0.866, *P* = 0.553; 45–54 years: β = 1.031, *P* = 0.664; ≥55 years: β = 1.290, p = 0.001), (D) serum GFAP levels and EDSS in NMOSD patients (≤44 years: β = 1.222, *P* = 0.005; 45–54 years: β = 1.149, *P* = 0.020; ≥55 years: β = 1.025, *P* = 0.722). MS, multiple sclerosis; NMOSD, neuromyelitis optica spectrum disorders.

To increase the number of patients evaluated in each group, we established three age groups (≤ 44, 45–54, and ≥55 years)^22^, ^23^, ^24^ (Table 4); 71 (39.4%), 54 (30.0%), and 55 (30.6%) patients were allocated to each age group, respectively. In MS patients, both NfL and GFAP levels tended to increase with EDSS scores in all age groups (Fig. 2A and B). The degrees of these correlations between serum markers and EDSS scores did not differ significantly among the age groups. However, in NMOSD patients, correlations between serum markers and EDSS scores were significantly different among the age groups (Fig. 2C and D). In particular, GFAP levels showed significantly stronger positive correlations with EDSS scores in the youngest, rather than the oldest, group (Pearson r, ≤ 44 years vs. ≥ 55 years, 0.754 vs. 0.075, p = 0.044 (corrected, Bonferroni)), whereas NfL levels exhibited opposite trends, with the strongest positive associations with EDSS scores seen in the oldest group (Pearson r, ≤44 years vs. ≥55 years, –0.182 vs. 0.594, p = 0.068 (corrected, Bonferroni)).

**Table 4:** Correlations between serum markers and Expanded Disability Status Scale scores according to age.

Three age groups	MS				NMOSD				p^†^ (MS vs. NMOSD)	
	Pearson r	n	*P* ^‡^		Pearson r	n	*P* ^‡^		Uncorrected	Corrected (Bonferroni)
			Uncorrected	Corrected (Bonferroni)			Uncorrected	Corrected (Bonferroni)		
Log NfL										
≤44	0.422**	56			‐0.182	13			0.066	0.198
45−54	0.301	32	0.546	>0.999	0.103	20	0.471	>0.999	0.498	>0.999
≥55	0.327	25	0.663	>0.999	0.594**	28	0.034	0.068	0.590	>0.999
Log GFAP										
≤44	0.196	56			0.754**	12			0.030	0.090
45−54	0.396*	31	0.346	0.692	0.530*	19	0.347	0.694	0.585	>0.999
≥55	0.302	25	0.656	>0.999	0.075	25	0.022	0.044	0.433	>0.999

EDSS, Expanded Disability Severity Scale; GFAP, glial fibrillary acidic protein; MS, multiple sclerosis; NfL, neurofilament light chain; NMOSD, neuromyelitis optica spectrum disorders.

Significant correlations with EDSS score,

* *P* < 0.05,

** *P* < 0.01,

*** *P* < 0.001;

^†^ *P* value for differences in correlation coefficients between the MS and NMOSD groups;

^‡^ *P* value for differences in correlation coefficients between the age groups within the disease group (vs. the reference [the youngest] group).

In the analyses with the two age groups stratified by the median age, the findings were also similar (Supplementary Table S1 and Supplementary Figure S1). Among MS patients, correlations between NfL (or GFAP) levels and EDSS scores were comparable between the age groups (Pearson r, <47 years vs. ≥47 years, NfL: 0.403 vs. 0.275, p = 0.457; GFAP: 0.193 vs. 0.360, *P* = 0.355). Among NMOSD patients, correlations between GFAP levels and EDSS scores tended to be closer in younger, rather than older, patients (Pearson r, <47 years vs. 47 years, 0.675 vs. 0.273, p = 0.041; Supplementary Figure S1D); those between NfL levels and EDSS scores (Pearson r, 47 years vs. ≥47 years, –0.171 vs. 0.518, p = 0.016; Supplementary Figure S1C) were also significantly different between the age groups.

Finally, because the period between the last attack and blood sampling was not comparable between patients with MS and NMOSD, we conducted age‐group analyses involving only patients who had experienced clinical attacks within the previous 5 years. In these patients, the interval between the last attack and blood sampling was similar between the diseases (MS [n = 65] vs. NMOSD [n = 48], median days [interquartile range], 475 [101—835] vs. 402 [84—932], *P* = 0.866). The age‐group analyses in these patients also showed results similar to those in all participants; the degree to which serum markers reflect disease severity tended to differ between the age groups in NMOSD, but not MS, patients (Table 5 and Supplementary Table S2).

**Table 5:** Correlations between serum markers and Expanded Disability Status Scale scores according to age in only patients who had clinical attacks within the previous 5 years

Three age groups	MS				NMOSD				*P* ^†^ (MS vs. NMOSD)	
	Pearson r	n	*P* ^‡^		Pearson r	n	*P* ^‡^		Uncorrected	Corrected (Bonferroni)
			Uncorrected	Corrected (Bonferroni)			Uncorrected	Corrected (Bonferroni)		
Log NfL										
≤44	0.386*	42			−0.182	13			0.095	0.285
45−54	0.300	12	0.792	>0.999	0.182	16	0.382	0.764	0.772	>0.999
≥55	0.495	11	0.727	>0.999	0.500*	19	0.069	0.138	0.988	>0.999
Log GFAP										
≤44	0.133	42			0.754**	12			0.022	0.066
45−54	0.485	12	0.285	0.570	0.567*	15	0.442	0.884	0.797	>0.999
≥55	0.379	11	0.495	0.990	−0.113	16	0.012	0.024	0.254	0.762

EDSS, expanded disability severity scale; GFAP, glial fibrillary acidic protein; MS, multiple sclerosis; NfL, neurofilament light chain; NMOSD, neuromyelitis optica spectrum disorders.

Significant correlations with EDSS score,

* *P* < 0.05,

** *P* < 0.01,

*** *P* < 0.001;

^†^ *P* value for differences in correlation coefficients between the MS and NMOSD groups;

^‡^ *P* value for differences in correlation coefficients between the age groups within the disease group (vs. the reference [the youngest] group).

## Discussion

In this study, we found that serum GFAP levels increased with age in patients with either MS or NMOSD, while serum NfL levels showed significant positive correlations with age in NMOSD only. Although serum GFAP levels were significantly higher in NMOSD than in MS patients, both serum NfL and GFAP levels demonstrated significant positive correlations with EDSS scores in both diseases. Notably, the degrees of correlations between serum markers and disease severity in MS were similar across the age groups, but those in NMOSD were significantly different among the age groups.

NfL and GFAP are cytoskeletal scaffolding proteins in neurons and astrocytes, respectively, which are released into the extracellular space following neuroaxonal or glial injury.^25^, ^26^ Because these proteins can be measured from body fluids including CSF and blood, they have been suggested as a promising biomarker in CNS demyelinating diseases. Higher levels of NfL were reported to be associated with high disease activity (recent relapses) and severity (higher EDSS scores), and to predict poor prognosis (conversion to MS after optic neuritis or clinically isolated syndrome).^27^, ^28^ NfL was even suggested as a marker to reflect treatment responses, as immunomodulatory agents significantly decreased NfL levels along with inflammation during treatment.^29^, ^30^, ^31^ GFAP levels were also reported to be related with disease activity and severity in these patients.^4^, ^7^ In addition, a possibility has been raised that GFAP levels could discriminate anti‐AQP4 antibody‐seropositive NMOSD from other demyelinating diseases such as anti‐MOG antibody‐associated diseases.^32^, ^33^ However, although fluid levels of these proteins are prone to be affected by aging and neurodegeneration, this variability has received little attention. This is the first study to analyze the clinical value of serum levels of these proteins according to patient age in CNS demyelinating diseases.

Our results confirm the clinical value of these proteins as a biomarker, showing that serum levels of NfL and GFAP were both closely correlated with disease severity. Serum NfL levels may plausibly correlate with increasing EDSS scores, because both MS and NMOSD are accompanied by damage to neuronal axons.^1^, ^34^, ^35^ Meanwhile, the relationship between GFAP levels and EDSS scores warrants discussion. Astroglial activation has been implicated in inflammatory demyelinating diseases in two aspects: (a) it may trigger immune‐system cascades, resulting in neurodegeneration, or (b) it may be involved in terminating immune responses, thus promoting remyelination.^26^ However, the close relationship between MS disease activity and GFAP levels in the CSF has been controversial.^35^, ^36^, ^37^ These uncertain results may have been due to the diagnostic insensitivity of CSF GFAP assays. Serum GFAP levels may be more sensitive than CSF GFAP levels in reflecting astroglial damage^4^ because GFAP expression occurs mainly in astrocyte branches, which constitute the blood–brain barrier. GFAP might preferentially drain into the blood rather than into the CSF.^4^

Both serum NfL and GFAP levels were also likely to increase with age. These positive associations may reflect the presence of age‐related neurodegeneration.^11^, ^13^, ^14^ However, the positive NfL‐age correlations in MS patients were not statistically significant and were substantially weaker than those in NMOSD patients. Although positive correlations between NfL levels and age in MS have been suggested,^6^, ^8^ the association was denied in a recent meta‐analysis that evaluated 10,059 individuals with neurological disorders.^38^ In the cited meta‐analysis, while healthy controls revealed significant positive correlations between age and NfL levels, patients with progressive diseases including fronto‐temporal dementia, amyotrophic lateral sclerosis, parkinsonian syndromes, and MS did not.^38^ These data suggest that neuropathological processes in progressive diseases may cause a plateau in NfL levels or mask age associations.^38^ Conversely, NMOSD, which lacks progressive phase,^1^ may have accentuated the positive relationship of NfL levels with age.

The clinical implications of the serum markers were different between MS and NMOSD, depending on patient age. In MS patients, the degrees of correlation between NfL (or GFAP) levels and disease severity were similar among age groups, suggesting that neuronal and astrocytic damage may occur at similar degrees throughout patients’ lifetime. These findings may reflect the steady and progressive disease processes, independent of relapsing events, in MS.^1^ On the contrary, in NMOSD patients, serum GFAP levels in the younger group showed stronger positive associations with disease severity than those in the older group. The high serum GFAP levels detected in patients with NMOSD may arise from immune‐mediated cellular cytotoxicity targeting the AQP4 water channel, which is densely expressed in astrocytes.^39^, ^40^, ^41^ These pathogenic processes may result in close correlations between GFAP levels and disease severity.^36^ With aging, however, the associations between GFAP levels and disease severity may become complicated and less specific. Along with neurodegeneration (i.e., increasing neuronal damage), secondary changes such as mixed changes in cortical GFAP‐positive astrocytes (decreases seen in layer I but increases seen in layers II–IV) and increased astrogliosis (i.e., increased extent of GFAP in the brain) in chronic lesions develop in patients with NMOSD;^26^, ^35^ these changes may weaken the degrees of correlation between GFAP and EDSS scores. Conversely, correlations between NfL levels and EDSS scores were not significant in younger patients but were prominent in older patients with NMOSD. Taken together, although astrocytic damage may be characteristic of NMOSD, particularly in younger patients, other age‐related processes may also be involved and become important in older patients with NMOSD.

Could then serum GFAP levels be a specific biomarker for NMOSD? We regard that the answer may also depend on patient age. In younger patients without age‐related neurodegeneration, serum GFAP levels may be a specific biomarker for NMOSD rather than for MS, while in older patients with advanced neurodegeneration, the specificity of GFAP as a diagnostic marker may be compromised. In old patients, serum levels of GFAP, which can increase with neurodegeneration and secondary changes, may reflect neurological disability in MS as well as NMOSD. Because the number of participants is small in this study, further larger studies to confirm this possibility are necessary.

The study limitations include the recruitment of individuals belonging to a single ethnicity (Korean) from a single center, which decreases the results’ general applicability. Additionally, because the number of patients in this study was relatively small, the degrees of correlations were not fully stratified according to the decade of age, leading to definition of the arbitrary age groups. We attempted to overcome this limitation by using two additional different age stratification criteria, and acquired consistent findings. MS and NMOSD patients had different clinical statuses at the time of blood sampling. These differences may have affected serum levels of Simoa markers and their clinical values. However, the results were reproduced even when we evaluated only the patients who had endured an attack within the previous 5 years to balance the intervals between the diseases. In addition, although we have adopted a dilution concentration according to the guideline and previous works,^6^, ^7^, ^8^, ^9^, ^21^ a possibility of a “hook effect,”^42^ diverging from the linear range of the standard curve, should be considered, because we used multi‐fold sample dilution in serum analyses. Therefore, the results should be interpreted with caution especially for high concentration values. Finally, the clinical implications of biomarkers were evaluated only in terms of correlations between serum levels and disease severity. Follow‐up data collected longitudinally to evaluate the clinical value of these biomarkers (e.g., predicting relapses and/or progressions) according to patient age are warranted.

In conclusion, although serum NfL and GFAP levels may reflect disease severity in both MS and NMOSD, the degrees to which these markers reflect disease severity differ significantly with patient age in NMOSD, but not in MS. These findings may indicate different pathogenic processes between MS and NMOSD, and suggest the need for different strategies to interpret biomarker data according to patient age and disease type.

## Author contributions

E.‐J.L, K.‐K.K., and Y.‐M.L were involved in conception and design of the study. All authors were involved in acquisition and analysis of data, and drafting manuscript and approval of the final draft.

## Conflict of interest

None.

## Supporting information


**Table S1** **.** Correlations between serum markers and Expanded Disability Status Scale (EDSS) scores according to two age groups, separated by the median age (47 years).
**Table S2** **.** Correlations between serum markers and Expanded Disability Status Scale (EDSS) scores according to two age groups, containing only patients who had experienced clinical attacks within the previous 5 years.
**Figure S1** **.** Associations between serum biomarkers and Expanded Disability Status Scale (EDSS) scores according to two age groups.
