# Supplementary material for: Clinical implication of serum biomarkers and patient age in inflammatory demyelinating diseases
Source: Ann Clin Transl Neurol. 2020 Jun 4;7(6):992–1001. doi: 10.1002/acn3.51070 (PMC7317646; doi:10.1002/acn3.51070)

**Supplementary Table 1.** Correlations between serum markers and Expanded Disability Status Scale (EDSS) scores according to two age groups, separated by the median age (47 years)

|  |  | **MS** | | |  | **NMOSD** | | |  | p† (MS vs. NMOSD) | |
| --- | --- | --- | --- | --- | --- | --- | --- | --- | --- | --- | --- |
|  |  | Pearson r | n | p‡ |  | Pearson r | n | p‡ |  | Uncorrected | Corrected (Bonferroni) |
| **Two age groups** | |  |  |  |  |  |  |  |  |  |  |
| Log NfL | < 47 | 0.403** | 63 |  |  | −0.171 | 17 |  |  | 0.043 | 0.086 |
|  | ≥ 47 | 0.275 | 50 | 0.457 |  | 0.518*** | 44 | 0.016 |  | 0.173 | 0.346 |
|  |  |  |  |  |  |  |  |  |  |  |  |
| Log GFAP | < 47 | 0.193 | 63 |  |  | 0.675** | 16 |  |  | 0.041 | 0.082 |
|  | ≥ 47 | 0.360* | 49 | 0.355 |  | 0.273 | 40 | 0.041 |  | 0.661 | >0.999 |

EDSS, Expanded Disability Severity Scale; GFAP, glial fibrillary acidic protein; MS, multiple sclerosis; NfL, neurofilament light chain; NMOSD, neuromyelitis optica spectrum disorders

Significant correlations with EDSS score, *p < 0.05, ** p < 0.01, *** p < 0.001

†p value for differences in correlation coefficients between the MS and NMOSD groups

‡p value for differences in correlation coefficients between the age groups within the disease group (vs. the reference [younger] group)

**Supplementary Table 2.** Correlations between serum markers and Expanded Disability Status Scale (EDSS) scores according to two age groups, containing only patients who had experienced clinical attacks within the previous five years

|  |  | **MS** | | |  | **NMOSD** | | |  | p† (MS vs. NMOSD) | |
| --- | --- | --- | --- | --- | --- | --- | --- | --- | --- | --- | --- |
|  |  | Pearson r | n | p‡ |  | Pearson r | n | p‡ |  | Uncorrected | Corrected (Bonferroni) |
| **Two age groups** | |  |  |  |  |  |  |  |  |  |  |
| Log NfL | < 47 | 0.370* | 46 |  |  | −0.171 | 17 |  |  | 0.068 | 0.136 |
|  | ≥ 47 | 0.253 | 19 | 0.658 |  | 0.467** | 31 | 0.038 |  | 0.430 | 0.860 |
|  |  |  |  |  |  |  |  |  |  |  |  |
| Log GFAP | < 47 | 0.138 | 46 |  |  | 0.675** | 16 |  |  | 0.031 | 0.062 |
|  | ≥ 47 | 0.436 | 19 | 0.262 |  | 0.223 | 27 | 0.085 |  | 0.456 | 0.912 |

EDSS, Expanded Disability Severity Scale; GFAP, glial fibrillary acidic protein; MS, multiple sclerosis; NfL, neurofilament light chain; NMOSD, neuromyelitis optica spectrum disorders

Significant correlations with EDSS score, *p < 0.05, ** p < 0.01, *** p < 0.001

†p value for differences in correlation coefficients between the MS and NMOSD groups

‡p value for differences in correlation coefficients between the age groups within the disease group (vs. the reference [younger] group)

**Supplementary Figure 1.** Associations between serum biomarkers and Expanded Disability Status Scale (EDSS) scores according to two age groups.

(A) Serum neurofilament light chain (NfL) levels and EDSS in MS patients (< 47 years: β = 0.182, p = 0.001; ≥ 47 years: β = 1.077, p = 0.053), (B) serum glial fibrillary acid protein (GFAP) levels and EDSS in MS patients (< 47 years: β = 1.054, p = 0.130; ≥ 47 years: β = 1.113, p = 0.011), (C) serum NfL levels and EDSS scores in NMOSD patients (< 47 years: β = 0.886, p = 0.512; ≥ 47 years: β = 1.220, p <0.001), (D) serum GFAP levels and EDSS in NMOSD patients (< 47 years: β = 1.192, p = 0.004; ≥ 47 years: β = 1.087, p = 0.088)

MS, multiple sclerosis; NMOSD, neuromyelitis optica spectrum disorders


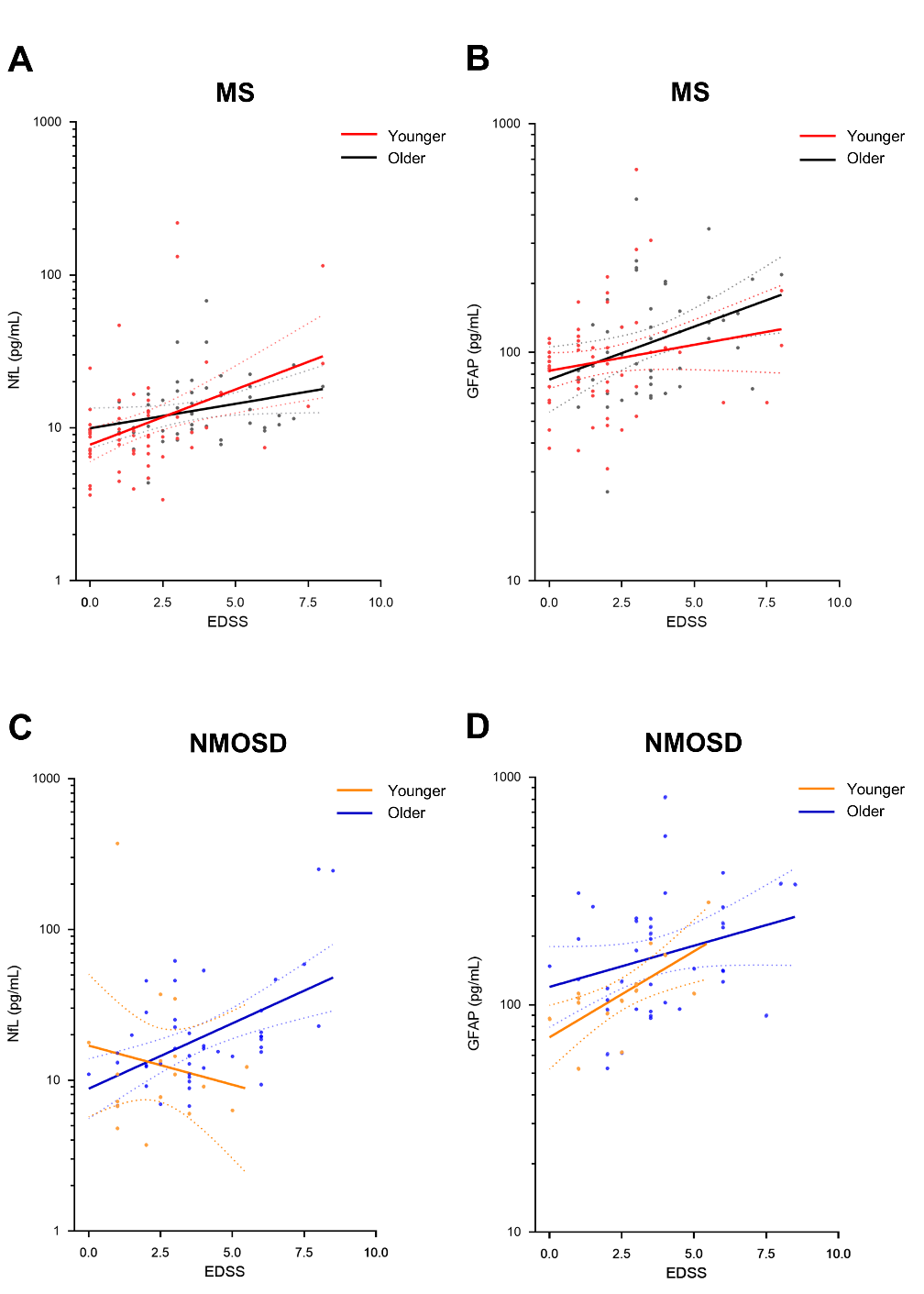

Supplement: Supplementary file 1 — Table S1 . Correlations between serum markers and Expanded Disability Status Scale (EDSS) scores according to two age groups, separated by the median age (47 years). Table S2 . Correlations between serum markers and Expanded Disability Status Scale (EDSS) scores according to two age groups, containing only patients who had experienced clinical attacks within the previous 5 years. Figure S1 . Associations between serum biomarkers and Expanded Disability Status Scale (EDSS) scores according to two age groups. [file ACN3-7-992-s001.docx]
